# Dietary phytogenics and galactomannan oligosaccharides in low fish meal and fish oil-based diets for European sea bass (*Dicentrarchus labrax*) juveniles: Effects on gut health and implications on *in vivo* gut bacterial translocation

**DOI:** 10.1371/journal.pone.0222063

**Published:** 2019-09-18

**Authors:** Silvia Torrecillas, Genciana Terova, Alex Makol, Antonio Serradell, Victoria Valdenegro, Elisabetha Gini, Marisol Izquierdo, Félix Acosta, Daniel Montero

**Affiliations:** 1 Grupo de Investigación en Acuicultura (GIA), IU-ECOAQUA, Universidad de Las Palmas de Gran Canaria, Crta. Taliarte s/n, Telde, Las Palmas, Canary Islands, Spain; 2 Department of Biotechnology and Life Sciences, University of Insubria, Via J.H. Dunant, Varese, Italy; 3 Delacon Biotechnik GmbH, Weissenwolffstrasse, Steyregg, Austria; 4 Biomar A/S. BioMar AS, POB 1282 Sluppen, Trondheim, Norway; University of Illinois at Urbana-Champaign, UNITED STATES

## Abstract

European sea bass were fed four low FM/FO (10%/6%) diets containing galactomannan oligosaccharides (GMOS), a mixture of garlic oil and labiatae plants oils (PHYTO), or a combination of both functional products (GMOSPHYTO) for 63 days before exposing the fish to an intestinal *Vibrio anguillarum* infection combined with crowding stress. In order to evaluate functional diets efficacy in terms of gut health maintenance, structural, cellular, and immune intestinal status were evaluated by optical and electron microscopy and gene expression analyses. A semi-automated software was adapted to determine variations in goblet cell area and mucosal mucus coverage during the challenge test. Feeding with functional diets did not affect growth performance; however, PHYTO and GMOS dietary inclusion reduced European sea bass susceptibility to *V*. *anguillarum* after 7 days of challenge testing. Rectum (post-ileorectal valve) showed longer (p = 0.001) folds than posterior gut (pre-ileorectal valve), whereas posterior gut had thicker submucosa (p = 0.001) and higher mucus coverage as a result of an increased cell density than rectum. Functional diets did not affect mucosal fold length or the grade of granulocytes and lymphocytes infiltration in either intestinal segment. However, the posterior gut fold area covered by goblet cells was smaller in fish fed GMOS (F = 14.53; p = 0.001) and PHYTO (F = 5.52; p = 0.019) than for the other diets. PHYTO (F = 3.95; p = 0.049) reduced posterior gut goblet cell size and increased rodlet cell density (F = 3.604; p = 0.068). Dietary GMOS reduced submucosal thickness (F = 51.31; p = 0.001) and increased rodlet cell density (F = 3.604; p = 0.068) in rectum. Structural TEM analyses revealed a normal intestinal morphological pattern, but the use of GMOS increased rectum microvilli length, whereas the use of PHYTO increased (p≤0.10) *Ocln*, *N-Cad* and *Cad-17* posterior gut gene expression. After bacterial intestinal inoculation, posterior gut of fish fed PHYTO responded in a more controlled and belated way in terms of goblet cell size and mucus coverage in comparison to other treatments. For rectum, the pattern of response was similar for all dietary treatments, however fish fed GMOS maintained goblet cell size along the challenge test.

## Introduction

Fish intestinal mucosal surface is thought to be a potential route of entrance for pathogenic bacteria [1] and consists of: (a) an extrinsic barrier, (b) a physical barrier and (c) a sub-epithelial or immunological defense barrier [2]. The mucosal extrinsic barrier constitutes the first line of defense against potential pathogens [3], protecting and lubricating the physical barrier and facilitating transport between the luminal contents and the epithelial cells [2,4].

Fish intestinal mucus is produced by goblet cells and is composed of a matrix of mucin glycoproteins and several humoral immune factors, such as immunoglobulins, complement proteins, C-reactive protein, lectins, lysozyme, proteolytic enzymes, and antimicrobial peptides [5]. Mucus granules are stored apically in the goblet cells and secreted at a slow baseline rate to maintain the mucus layer over the epithelial barrier [6–8].

The second line of the intestinal mucosal defense system is constituted by a simple epithelial layer composed of intestinal epithelial cells (IECs) sealed among them by the lateral surface via tight juntions (TJs), adherens junctions (AJs), and desmosomes, also including scattered goblet cells and rodlet cells. TJs are located at the apical end of the lateral side of IECs and play a pivotal role in preserving intestinal permeability. TJs regulate nutrients, toxins, antigens, and microbes trafficking through the paracellular channels, control proliferation and transcription signals, link proteins to the filamentous cytoskeleton, and maintain cellular polarity [9]. AJs are formed by clusters of cadherin molecules, and are immediately localized below TJs; their main function is to mediate strong cell-cell adhesion. Similarly, desmosomes provide stronger adhesion and intercellular communication between IECs [10].

Goblet cell density, intestinal mucus composition and its discharge rates, as well as IEC structure and adhesion may vary in response to nutritional, physiological, immunological, and/or microbiological factors. Moreover, despite the fact that the extrinsic and physical barriers together being extremely operational in preventing contact among detrimental substances and fish gut-associated lymphoid tissue (GALT), it is not possible to completely prevent contact [11]. In fact, an exchange is desirable in order to establish tolerance to autochthonous microbiota. GALT comprises a unique array of innate and adaptive immune cells and molecules that act in concert to protect the host against pathogens [12]. Fish GALT mainly contains intraepithelial lymphocytes (IELs), eosinophil intraepithelial granulocytes/mast cells (EGCs/MCs), *lamina propria* lymphocytes/granulocytes, and macrophages, populations and distribution also possibly varying upon an inflammation-like status.

An inflammatory gut reaction can be induced by a variety of factors, such as infection, stress or changes in feed composition [13–16]. In this latter perspective, the rational use of limited marine raw materials *via* replacements by vegetable meals (VM) and oils (VO) in feeds for marine fish species is associated with variable side effects on fish gut health [17–24]. For European sea bass (*Dicentrarchus labrax*), in particular, feeding low fishmeal (FM) and fish oil (FO) dietary content (10–5%FM/6-3%FO) results in a posterior gut inflammation-like status characterized by: a swelling of the *lamina propria* and submucosa, an increased density of goblet cells, an intestinal up-regulation of several inflammation related genes and altered microbiota populations [23]. In this case, a proper description of the intestinal morphological and functional patterns arising from feeding diets with a high VM/VO content will provide a better understanding of the underlying mechanisms, helping to predict the course of inflammation, and serve as a guide for remedial interventions [25].

Accordingly, the use of functional additives simultaneously with a low FM/FO based diet may help to buffer possible negative side effects on gut health. Prebiotic fibers and phytogenics have been proposed as effective candidates to immunomodulate fish through the diet. Among them, rich GMOS products obtained from different sources and phytogenics have been associated with enhanced fish production performance and health status [26–32].

Thus, the aim of the present study was to determine the effects of GMOS, a phytogenic (mixture of garlic oil and labiatae plant extracts) and a combination thereof on European sea bass juvenile’s disease resistance against an experimental intestinal *Vibrio anguillarum* infection combined with stress by confinement in relation to posterior gut and rectum status when supplemented with low FM and FO diets. For that purpose, structural, cellular, and immune intestinal dietary-associated alterations were evaluated by gene expression analyses and by optical and electron microscopy studies, using a semi-automated software adapted to determine variations in the goblet cell area and percentage of mucosal surface covered by mucus along the experimental intestinal *Vibrio anguilarum* infection.

## Materials and methods

### Ethics statement

Animal manipulation during these experiments complied with the guidelines of the European Union Directive (2010/63/EU) and Spanish legislation (RD 53/2013) for animal experiments. The Bioethical Committee of the University of Las Palmas de Gran Canaria approved all the protocols performed in the present study (approval n. 007/2012 CEBA ULPGC). Fish handling was performed under natural clove oil anesthesia (0.2 mL/L; Guinama S.L; Spain, Ref. Mg83168), and discorfort, stress and pain to the experimental animals was avoided, as much as possible, along the experiment. For sampling, fish were euthanized with and overdose of natural clove oil (5mL/L; Guinama S.L; Spain, Ref. Mg83168).

### Diets

Four experimental diets were prepared consisting of 10% FM and 6% FO and containing different additives: galactomannan oligosaccharides (GMOS; Delacon, Austria), a mixture of garlic and labiatae-plants oils (PHYTO; Delacon, Austria), and a combination of the two additives (GMOSPHYTO). The levels of GMOS (5000 ppm), PHYTO (200 ppm), and GMOSPHYTO (5200 ppm) were chosen according to commercial recommendations (Delacon, Austria). Diets were isoenergetic and isonitrogenous, covered all known nutritional requirements for sea bass (*Dicentrarchus labrax*), and were manufactured by an extrusion process in the BioMar Tech-Centre (Brande, Denmark). To ensure product stability GMOS was included in the diet in the mix pre extrusion process and replacing standard carbohydrates, PHYTO was included post extrusion process by vaccum coating and homogenized with the dietary fish oil. The stability of the phytogenic was evaluated previous to diet production, after production and at the beginning of the feeding trial. Diet ingredients, analyzed proximate composition, and fatty acid profiles are detailed in Tables 1 and 2.

**Table 1:** Main ingredients and analyzed proximate composition of the diets.

	Diets			
Ingredients (%)	CONTROL	GMOS	PHYTO	GMOSPHYTO
Fish meal^1^	10	10	10	10
Soya protein concentrate	18.9	18.9	18.9	18.9
Soya Meal	12.0	12.0	12.0	12.0
Corn gluten meal	25.0	25.0	25.0	25.0
Wheat	8.7	8.2	8.7	8.2
Wheat gluten	2.0	2.0	2.0	2.0
Guar Meal	8.0	8.0	8.0	8.0
Rapeseed extracted	3.0	3.0	3.0	3.0
Fish oil^2^	6.7	6.7	6.7	6.7
Rapeseed oil^3^	5.4	5.4	5.4	5.4
Vitamin and mineral premix^4^	3.7	3.7	3.7	3.7
Antioxidant ^5^	0.06	0.06	0.06	0.06
Galactomannan oligosaccharides^6^	0	0.5	0	0.5
Phytogenic^7^	0	0	200ppm	200ppm
**Proximate composition (% of dry matter)**				
Crude lipids	19.91	20.44	20.47	20.72
Crude protein	49.30	49.27	49.76	49.85
Moisture	5.10	5.01	5.06	5.17
Ash	7.02	6.41	6.49	6.39
Gross Energy (MJ/kg, as is)	22.07	22.11	22.17	22.25

^1^ South-American, Superprime 68%.

^2^ South American fish oil.

^3^ DLG AS, Denmark.

^4^ Vilomix, Denmark.

^5^ BAROX BECP, Ethoxyquin.

^6^Delacon Biotechnik GmbH, Austria.

^7^Delacon Biotechnik GmbH, Austria.

**Table 2:** Fatty acid composition (% of total identified fatty acids) of the experimental diets.

	CONTROL	GMOS	PHYTO	GMOSPHYTO
**14:0**	3.28	2.91	3.02	2.92
**14:1n-5**	0.03	0.02	0.02	0.02
**14:1n-7**	0.13	0.12	0.12	0.12
**15:0**	0.27	0.24	0.25	0.25
**15:1n-5**	0.02	0.02	0.02	0.02
**16:0ISO**	0.05	0.04	0.05	0.05
**16:0**	13.45	12.58	13.03	12.95
**16:1 n-7**	3.46	3.27	3.35	3.27
**16:1n-5**	0.14	0.13	0.13	0.13
**16:2n-6**	0.00	0.00	0.00	0.01
**16:2n-4**	0.29	0.30	0.31	0.30
**17:0**	0.28	0.31	0.31	0.29
**16:3n-4**	0.17	0.14	0.16	0.14
**16:3n-3**	0.08	0.07	0.07	0.08
**16:3n-1**	0.04	0.04	0.03	0.03
**16:4n-3**	0.36	0.40	0.37	0.35
**16:4 n-1**	0.00	0.00	0.00	0.00
**18:0**	2.78	2.66	2.77	2.76
**18:1 n-9**	31.75	31.40	31.74	31.68
**18:1 n-7**	2.66	2.63	2.70	2.76
**18:1 n-5**	0.14	0.13	0.13	0.13
**18:2n-9**	0.08	0.04	0.04	0.04
**18:2 n-6**	17.09	18.98	18.81	19.17
**18:2n-4**	0.11	0.11	0.11	0.10
**18: 3n-6**	0.12	0.09	0.09	0.10
**18:3n-4**	0.08	0.07	0.07	0.06
**18:3 n-3**	4.53	4.66	4.56	4.53
**18:3n-1**	0.02	0.01	0.02	0.01
**18:4 n-3**	0.99	1.03	0.95	0.95
**18:4 n-1**	0.05	0.05	0.05	0.05
**20:0**	0.39	0.42	0.43	0.48
**20:1 n-9**	0.27	0.25	0.25	0.24
**20: 1n-7**	2.73	2.49	2.53	2.51
**20: 1n-5**	0.16	0.13	0.14	0.13
**20: 2n-9**	0.05	0.07	0.05	0.07
**20:2 n-6**	0.21	0.17	0.17	0.17
**20:3n-9**	0.02	0.02	0.02	0.03
**20:3 n-6**	0.05	0.05	0.05	0.05
**20:4 n-6**	0.36	0.37	0.37	0.36
**20: 3n-3**	0.07	0.06	0.06	0.06
**20:4 n-3**	0.29	0.32	0.29	0.29
**20:5 n-3**	4.39	4.82	4.45	4.38
**22:1 n-11**	3.04	2.82	2.85	2.82
**22:1 n-9**	0.37	0.33	0.33	0.33
**22:4 n-6**	0.05	0.05	0.05	0.05
**22:5 n-6**	0.03	0.04	0.04	0.03
**22:5 n-3**	0.59	0.63	0.59	0.59
**22:6 n-3**	4.46	4.52	4.07	4.14
**Ʃ Saturates**	20.51	19.16	19.85	19.69
**Ʃ Monoenes**	44.90	43.73	44.31	44.16
**Ʃ n-3**	15.75	16.51	15.40	15.37
**Ʃ n-6**	17.92	19.75	19.58	19.94
**Ʃ n-9**	32.54	32.10	32.44	32.39
**Ʃ n-3HUFA**	9.79	10.34	9.45	9.46
**Ʃ n-6HUFA**	0.50	0.51	0.51	0.50
**EPA+DHA**	8.85	9.33	8.52	8.52
**n-3/n-6**	0.88	0.84	0.79	0.77

### Experimental conditions

#### Experiment I: Feeding trial

Nine hundred European sea bass juveniles reared in a local farm (Aquanaria, Castillo del Romeral, Gran Canaria, Canary Islands, Spain) were transferred to the facilities of the Parque Científico-Tecnológico Marino (PCTM) at University of Las Palmas de Gran Canaria (Telde, Canary Island, Spain) and adapted during 4 weeks to facility water conditions (6.6–6.1 ppm dissolved O_2_, 18.2–20.2°C). Afterwards, fish were randomly distributed in 12 fiberglass tanks of 500 L (75 fish/tank) at an initial density of 3.5 kg·m^3^ (mean weight±SD: 23.5±0.8g; mean length±SD: 12.00±0.15cm) in an open water system with natural photoperiod (12L/12D). All groups were fed until apparent satiation 3 times a day, 6 days a week for 63 days. Growth performance was evaluated at the end of the feeding trial. For sampling, fish were caught and immediately euthanized with and overdose of natural clove oil (5mL/L; Guinama S.L; Spain, Ref. Mg83168). Samples of posterior gut and rectum for optical (n = 4 fish/tank) and electron morphology (n = 3 fish/tank) studies were also collected. For gene expression analyses, posterior gut of 6 fish per diet was excised, washed in diethyl pyrocarbonate (DEPC) water, placed into RNAlater^™^ (Sigma-Aldrich, Sant Louis, MO, USA) and stored at 0°C until RNA extraction.

#### Experiment II: Intestinal infection and stress challenge

After 63 days of feeding, 45 anesthesized fish/diet (0.2 mL/L natural clove oil) from Experiment I were transported pooled from the on-growing facilities of the PCTM-ULPGC (Telde, Canary Islands, Spain) to the Marine Biosecurity (MBS) facility situated in the same PCTM-ULPGC and exposed to an intestinal bacterial challenge combined with a confinement stress panel for 7 days. Anesthesized fish were infected by intestinal inoculation of *Vibrio anguillarum* (10^5^ cfu·ml^-1^ per fish; strain 507, isolated from a clinical outbreak in Canary Islands), as described in ref. [33]. The dose inoculated was previously determined in similar dietary and culture conditions by gradient of bacterial concentration. The dose inoculated was selected by common linerazitation of sigmoidal curves. Immediately after inoculation and still anesthesized, fish were subjected to a confinement stressor by increasing the feeding trial culture density by ten fold (final challenge density = 35 kg/m^3^) in small cages (15 fish/cage; 3 cages/diet). For sampling, fish were caught and inmediately euthanized with and overdose of natural clove oil (5mL/L; Guinama S.L; Spain, Ref. Mg83168). Samples of posterior gut and rectum (n = 6 fish/diet) were taken for optical microscopy at 2h, 24h, and 7 days of confinement. For the duration of the experiment, fish were fed with their respective diets three times a day. Naturally dead fish were collected, necropsied and *V*. *anguillarum* confirmed as the causative agent of death recorded by standard biochemical procedures. Relative percent survival (RPS) was calculated as described in ref. [34], following the equation: RPS = [1- Mortality of fish fed supplemented diet (%) / Mortality of fish fed control diet (%)] * 100.

### Biochemical composition of diets

Feed biochemical composition was analyzed according to standard procedures [35]. Dry matter content was determined after drying in an oven (110ºC) to constant weight and ash content by combustion in a muffle furnace (600°C, 12h). Crude lipid was extracted as described in ref. [36] and crude protein content (Nx6.25) by using the Kjeldahl method. Fatty acid methyl esters were obtained by transmethylation with 1% sulfuric acid in methanol as described in ref. [37] and separated by gas chromatography (GC-14A, Shimadzu, Japan). A GC Supercolovax-10-fused silica capillary column (Supelco, Bellefonte, USA) was used for the separation with helium as a carrier gas, applying the conditions described in ref. [38]. Fatty acid methyl esters were quantified by flame ionizator detector and identified by comparing them with external and well-characterized fish oils standards (EPA 28, Nippai, Ltd. Tokyo, Japan).

### Morphological studies

**Fig 1:**
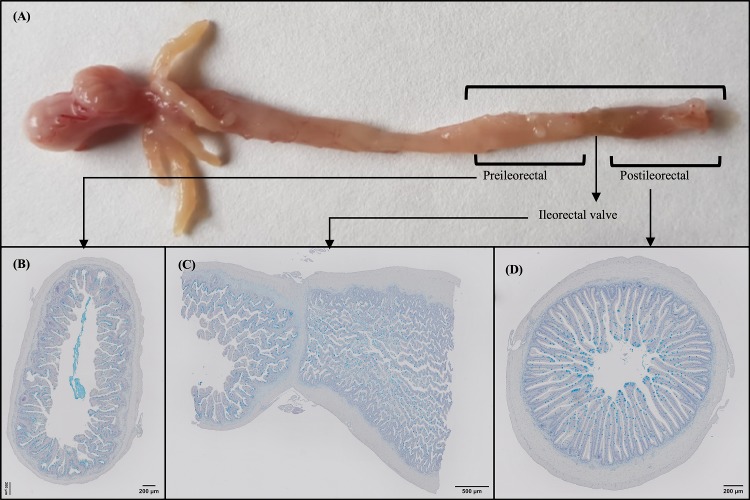
Nomenclature used for the gut segments used in this study. (A) Gut of European sea bass (*Dicentrarchus labrax*) juvenile, posterior gut (preileorectal valve segment) and rectum (postileorectal valve segment) regions sampled for morphological, morphometric, and immunohistochemical analyses. (B) Detailed micrograph of posterior gut stained with Alcian Blue (pH = 2.5); observe the shorter folds and higher density of goblet cells. Scale bar 200 μm; (C) Detailed micrograph of the ileorectal valve separating European sea bass posterior gut and rectum. Scale bar 500 μm. (D) Detailed micrograph of European sea bass rectum stained with Alcian Blue (pH = 2.5); observe the longer and thinner folds, the lower density of goblet cells, and the wider muscular layer. Scale bar 200 μm.

Fish posterior gut and rectum were dissected out and separated as pre-ileorectal valve and post-ileorectal valve segments as detailed in Fig 1. From each segment, three to six transverse sections (N_feeding_ = 12 fish per diet; N_challenge_ = 6 fish per diet and sampling point) were taken and fixed at 4°C in 4% paraformaldehyde. After 48 hours, samples were dehydrated and embedded in paraffin. Sections of 4μm were stained with hematoxylin and eosin (H&E) for optical examination, with Alcian Blue (pH = 2.5) in order to differentiate goblet cell secreting acid mucins, and with May-Grünwald/Giemsa (MGG) for studing leukocyte populations distribution [39]. Afterwards, slides were digitally scanned in a digital scanner Olympus VS120 (Optic system BX61VS, Tokyo, Japan) equipped with VC50 and VS-XM10 cameras and acquired with Olympus VS software (VS-NIS-SQL-V2.6, Tokyo, Japan).

#### Posterior gut and rectum morphometrical and mucus production studies

For posterior gut and rectum morphometry H&E/MGG-stained sections were used (N_feeding_ = 12 fish per diet; N_challenge_ = 6 fish per diet and sampling point). Mucosal fold height, width, and submucosal thickness were analyzed using the measurement tool of the image analysis program cellSens Dimension Desktop 1.16 (Olympus Iberia, Spain). The measurements of each variable were replicated 45 times for each fish. Two scientists unaware of the experimental treatments evaluated separately the cellular infiltration level of eosinophilic granulocytes (ECGs) and lymphocytes in both the *lamina propria* within the intestinal folds and the submucosa on the digitalized images. The scientists used MGG-stained sections and assigned a histological score (on a range from 0–3) to each feature based on a previously established histological scoring system [8]. The score 0 was assigned for no observed infiltration, 1 for low, 2 for moderate, and 3 for high infiltration.

For determining of mucus production related parameters, an Alcian Blue (pH = 2.5) section was used. CellSens Dimension Desktop 1.16 (Olympus Iberia, Spain) was specifically calibrated to determine the number of goblet cells, goblet cell area (μm^2^), goblet cell minimum diameter and perimeter (μm), and the % of mucosal surface (fold) covered by mucus (%) in a in a semi-automated way for each intestinal section.

#### Posterior gut and rectum structure study

Fish intestines were dissected out; the posterior gut and rectum separated as pre-ileorectal valve and post-ileorectal valve segments (n = 9 dietary treatment) were cut in small pieces and immediately fixed at 4°C in 2.5% glutaraldehyde in 0.15M HEPES buffer (pH = 7.4), rinsed in HEPES buffer, post fixed at 4°C in 2% osmium tetroxide and 2% uranyl acetate, dehydrated in graded ethanol series, and embedded individually in an Embed 812 (Electron Microscopy Sciences (EMS), PA, USA) resin block. Semithin (1 μm) serial transverse sections (N = 3 per individual fish) were contrasted with toluidine blue and examined under light microscopy [40]. Ultrathin (50 nm) sections were contrasted with lead citrate and examined with a JEOL JEM-1011 Transmission Electron Microscope (TEM; JEOL USA, Inc, USA) equipped with a digital camera MegaView III soft imaging system CCD Camera (EMSIS GmbH, Germany). Scientists who were unaware of the experimental treatments qualitatively evaluated the intestinal membrane lining appearance, cytoplasmatic electrondensity, enterocytes packaging, TJ structure, and level of infiltrated leukocytes. Microvilli length and cell dimensions were measured with cellSens Dimension Desktop 1.16 (Olympus Iberia, Spain).

### Gene expression analysis

#### Total RNA extraction and cDNA synthesis

Total RNA was extracted from fish gut using an automatic system (Maxwell® 16 Instrument, Promega) and a total RNA purification kit (Maxwell® 16 LEV simplyRNA Tissue). The RNA was quantified by using NanoDrop^™^ spectrophotometer (Thermo Scientific, Italy) and reverse transcribed into cDNA following the protocol of the SuperScript III Reverse Transcriptase kit (Invitrogen, Milan, Italy).

#### Primer design, amplification, and molecular cloning and sequencing of 7 target genes

The primers used for the amplification of *Ocln*, *ZO-1*, *Cad-17*, *E-Cad*, *N-Cad*, *α-Tub*, and *Vim* genes are reported in Table 3. They were designed based on the orthologous sequences of *Salmo salar* (Genbank acc. nr. **XM_014123589.1**), *Sparus aurata* (**KF861990.1**), and *Fundulus heteroclitus* (**XM_021308001.1***)* for *Ocln*; *S*. *aurata* (**KF861994.1**), and *Maylandia zebra* (**XM_004567665.3**) for *ZO-1*; *S*. *aurata* (**KF861996.1**), *F*. *heteroclitus* (**XM_021308835.1**), and *Oreochromis niloticus* (**XM_003450934.3**) for *Cad-17*; *S*. *aurata* (KF861995.1), *O*. *niloticus* (XM_019357299.1), and *Oncorhynchus mykiss* (AB787267.1) for *E-Cad* gene; *F*. *heteroclitus* (**XM_012875420.2**), *O*. *niloticus* (**XM_005476357.3**), *S*. *salar* (**XM_014123187.1**), and *Labrus bergylta* (**XM_020635352.1**) for *N-Cad*; *Dicentrarchus labrax* (AY326429.1) for *α-TUB*; *S*. *aurata* (**KF857332.1**), *S*. *salar* (**NM_001140475.1**), and *O*. *mykiss* (**NM_001124729.1**) for *Vim* gene.

**Table 3:** Primers used for the molecular cloning of the target genes.

Gene	Primer	Nucleotide sequence (5’- 3’)
**Ocln**	Forward	CATGGTGTGTGGATTCCTGGT
	Reverse	CGTCTCTTGCCCCTGTTGG
**ZO-1**	Forward	GGCCATGAAACCGCAGTCAG
	Reverse	ATCTTTTCTCCACTGGGCTCAC
**Cad-17**	Forward	AGAGGAGCTGGACAGAGA
	Reverse	CAGAGGCAGCTCATTGTTGA
**E-Cad**	Forward	TACACTGTGGTCCTGAGGGT
	Reverse	GTGCTGTCGGGTCATTGTCA
**N-Cad**	Forward	CGAACGCCATCAACATCAC
	Reverse	TAGACGGCGGATTCCCAC
**α-Tub**	Forward	AACTCCATCCTGACCACC
	Reverse	CCATCTGATTGGCTGGCTCA
**Vim**	Forward	TGCAGAGCTTCAGACAGGAT
	Reverse	GGCCATCTCGTCCTTCATGT

An aliquot of cDNA obtained by reverse transcription was amplified by PCR using the designed primer sets for each gene and GoTaq Green Master Mix (Promega, Milan, Italy), as described in ref. [41]. The plasmid was finally purified using the NucleoSpin® Plasmid kit (Macherey-Nagel Milan, Italy) and then sequenced in both directions (T7 and SP6).

### One-step TaqMan® real-time RT-PCR for mRNA copies quantification of 7 target genes

#### Generation of synthetic mRNAs

Based on the cDNA sequences of *D*. *labrax’s Ocln*, *ZO-1*, *Cad-17*, *E-Cad*, *N-Cad*, *α-Tub and Vim* genes, forward and reverse primers were designed for each gene (Table 4). The forward primers were designed to contain the sequence of the T3 or T7 RNA polymerase promoter necessary for *in vitro* transcription of the mRNAs of each target gene at the 5' end. The T3/T7 forward primers and their respective reverse primers were used in a conventional PCR reaction. The PCR product was then cloned and sequenced and its molecular weight was determined using the formula described in ref. [41]:

**Table 4:** Primers used for the synthesis of standard mRNAs.

Gene	Primer	Nucleotide sequence (5’- 3’)
**Ocln**	T7 Forward	taatacgactcactatagggTGGGTGAACAATGTGGAGGA
	Reverse	CGTCTCTTGCCCCTGTTGG
**ZO-1**	T7 Forward	taatacgactcactatagggCCATGAAACCGCAGTCAG
	Reverse	ACGGCGATCAAAGTAGGACA
**Cad-17**	T7 Forward	taatacgactcactatagggAGAGGAGCTGGACAGAGA
**E-Cad**	T3 Forward	caattaaccctcactaaaggg TACACTGTGGTCCTGAGGGT
	Reverse	GTGCTGTCGGGTCATTGTCA
	Reverse	CAGAGGCAGCTCATTGTTGA
**N-Cad**	T7 Forward	taatacgactcactatagggCGAACGCCATCAACATCAC
	Reverse	TAGACGGCGGATTCCCAC
**α-Tub**	T7 Forward	taatacgactcactatagggAACTCCATCCTGACCACC
	Reverse	CCATCTGATTGGCTGGCTCA
**Vim**	T7 Forward	taatacgactcactataggg TGCAGAGCTTCAGACAGGAT
	Reverse	GGCCATCTCGTCCTTCATGT

*In vitro* transcription was performed using T3 or T7 RNA polymerase and other reagents supplied in the RiboProbe *InVitro* Transcription System kit (Promega, Italy) according to the manufacturer’s protocol.

#### Generation of standard curves

The *in vitro* transcribed mRNAs of each target gene were used as quantitative standards t analyze experimental samples [41]. The sequences of primers and Taqman® probes used for RT-PCR quantification are shown in Table 5. Taqman® PCR reactions were performed on a Bio-Rad® CFX96^™^ System. The raw data of Taqman® PCR runs were collected by CFX^™^ Software.

**Table 5:** Primers and probes used for one-step Taqman real-time RT-PCR.

Gene	Primer	Nucleotide sequence (5’- 3’)
**Ocln**	Forward	GGACGAAGACGACAACAACGA
	Reverse	CCATGGGAGAAAGCCTCTGA
	Taqman Probe	TACTCAGAGAGAACCACGAGCCGGCC
**ZO-1**	Forward	CGGCCTGCAGATGTTCCTAA
	Reverse	GCTGAGGGAATTGGCTTTGA
	Taqman Probe	CCTGCGAGTGCACCTGGCCC
**Cad-17**	Forward	TGCCCACCTGACTTACATCATC
	Reverse	TTCCAGTGGCAGCATCAATG
	Taqman Probe	CCGGATGATTCAGCAACCAAAACCTTCT
**E-Cad**	Forward	CGGAGAGGATGATCAGGACTATG
	Reverse	TACTGTGGAGCTGGCATGAAGT
	Taqman Probe	TTCACCGTGGTCTGGACAACCGA
**N-Cad**	Forward	CGTGCTGCTGTTTGTGGTATG
	Reverse	CTCACATCATCCTCTGGATCGA
	Taqman Probe	AAGAACGTCAGGCGAAGCAGCTCCTC
**α-Tub**	Forward	AGGCTCATTGGCCAGATTGT
	Reverse	CAACATTCAGGGCTCCATCA
	Taqman Probe	TCTTCAATCACAGCCTCGCTTCGCT
**Vim**	Forward	GATGTCCGCCTGCAGTATGA
	Reverse	GGTGAGGTCAGCAAACTTGGA
	Taqman Probe	AACCTGGCCTCCAAAAACATCCATGAG

### Statistical analyses

Statistical analyses followed the methods described in ref. [42]. All data presented were tested for normality and homogeneity of variance. If necessary, data were transformed. Means and standard deviations (SD) were calculated for each parameter measured. Significant differences were considered when p≤0.05. Differences between dietary treatments were established by one-way ANOVA. When F values showed significance, individual means were compared using post hoc Tukey for multiple means comparison. The individual effects of prebiotic and phytogenic were analyzed by two-way ANOVA analyses, where GMOS and PHYTO were established as fixed factors. *P* values obtained for each evaluated parameter are reported in the corresponding results tables. Morphological and immunohistochemical findings, which were based on a range scale evaluation, were analyzed by paired comparisons among them (Mann-Whitney U test). Similarly, for morphometric analyses, posterior and rectum sections were compared by T student or Mann-Whitney U test, depending on the normality of the data. Analyses were performed using the SPSS Statistical Software System v21.0 (SPSS, Chicago, IL, USA) and PRIMER 7 with PERMANOVA complement (Auckland, New Zealand).

## 3. Results

### Experiment I: Feeding trial

#### Growth parameters and biometry

After 63 days of feeding, fish grew properly and presented a 2.6x increase in body weight along the 63 days of feeding, representing a relative growth (%) of a 158.8±16.3, however the utilization of GMOS, PHYTO, or a combination of both functional products did not induce differences (p>0.05) in fish growth or diet utilization, in terms of final body weight (59.0±1.8), total length (16.7±0.1), specific growth rate (1.5±0.1), or feed conversion ratio (1.2±0.1). The recorded mortality during the feeding trial was negligible (<1%) and not associated to an specific diet.

#### Posterior gut and rectum morphometrical and mucus production

**Fig 2:**
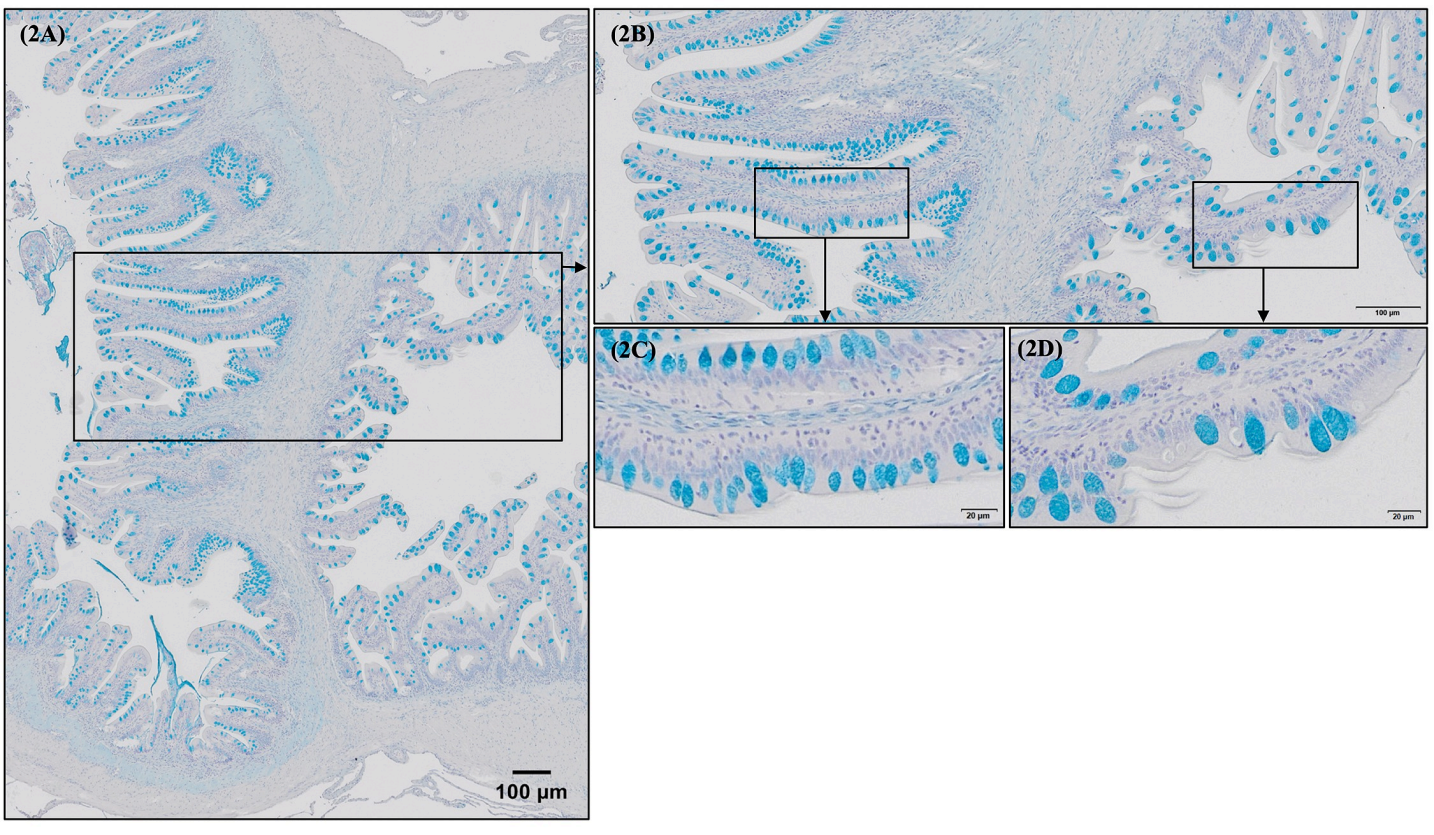
Morphological differences between European sea bass (*Dicentrarchus labrax*) posterior gut (preileorectal valve segment) and rectum (postileorectal valve segments) goblet cell size and distribution on mucosal surface. A similar morphology and mucus production pattern were observed in all the fish intestinal sections studied. A general overview of goblet cell distribution in posterior gut and rectum (separated by ileorectal valve) is detailed in Fig 2A and 2B (Alcian Blue, pH = 2.5; Bar 100 μm). Note the greater fold area covered by mucus in posterior gut compared to rectum as a result of increased cell density as posterior gut goblet cells are smaller than goblet cells located in rectum (Fig 2C vs 2D; Alcian Blue, pH = 2.5; Bar 20 μm).

**Fig 3:**
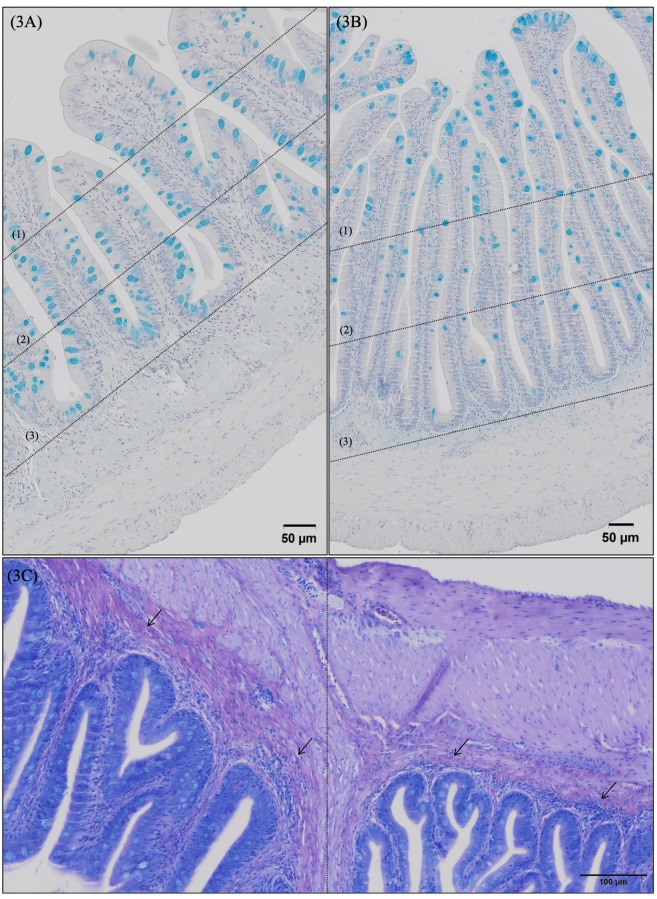
Detailed micrograph of posterior gut (3A) and rectum (3B) goblet cells distribution along the folds stained with Alcian Blue (pH = 2.5). Observe a higher density of goblet cells on posterior gut mid (2) and basal (3) fold regions compared to apical (1) fold region, whereas rectum presents greater goblet cell density on apical (1) and mid (2) regions in comparison to basal (3) zone. Scale bar 50 μm. (3C) Detailed micrograph of posterior gut (preileorectal valve; right) and rectum (postileorectal valve; left) stained with May-Günwald Giemsa; observe the wider submucosa (→) in posterior gut than in rectum segments. Scale bar 100 μm.

Morphological evaluation of H&E/MGG-stained sections of fish posterior gut and rectum segments showed a well-organized folding pattern, lack of cell debris, and an intact intestinal epithelial barrier for all the fish experimental groups. In general terms, by comparing posterior gut (preileorectal valve segment) and rectum (postileorectal valve segment), the latter presented longer (p = 0.001) folds than posterior gut (Table 6, Fig 1B vs 1D). In terms of mucus production, posterior gut presented a greater fold area covered by mucus as a result of an increased cell density (Table 6, Fig 2A and 2B), since posterior gut goblet cells were smaller (p = 0.001) than goblet cells located in rectum (Table 6, Fig 2C vs 2D). In addition, goblet cell distribution along the folds varied between intestinal segments. In European sea bass posterior gut, a higher density of goblet cells on the mid and basal fold regions was found than in the apical fold region (Fig 3A), whereas in European sea bass rectum goblet cell density was higher on the apical and mid regions than in the basal zone (Fig 3B). Furthermore, rectum segment presented a thinner submucosa (p = 0.001) than the posterior gut (Table 6, Fig 3C).

**Table 6:** Posterior intestine and rectum morphometric parameters for European sea bass (*Dicentrarchus labrax*) juveniles at the end of the feeding trial (63days).

	Dietary treatments				Two-way ANOVA			
	C	GMOS	PHYTO	GMOSPHYTO	GMOS	PHYTO	GMOS*PHYTO	Posterior vs Rectum
**Fold length (**μ**m)**								
Posterior gut	440.1±39.1	381.1±52.6	366.0±10.4	406.1±74.2	**NS**	**NS**	**NS**	p = 0.001
Rectum	668.4±59.4	645.1±19.9	645.9±66.2	706.5±51.0	**NS**	**NS**	**NS**	
**Fold area covered by mucus (%)**								
Posterior gut	10.3±0.2	8.8±1.5	9.2±0.9	8.7±2.4	**NS**	**NS**	**NS**	p = 0.001
Rectum	6.4±0.5	5.2±0.7	6.6±2.1	6.6±0.7	**NS**	**NS**	**NS**	
**Goblet cell area (**μ**m**^**2**^**)**								
Posterior gut	81.5±15.2a	61.3±10.3b	56.2±8.2b	66.2±10.2ab	**NS**	F = 3.95; p = 0.05	F = 8.73; p = 0.006	p = 0.001
Rectum	114.5±4.0a	86.8±20.4b	96.8±26.7ab	109.6±8.4ab	**NS**	**NS**	F = 10.05; p = p = 0.003	
**Goblet cell minimum diameter (**μ**m)**								
Posterior gut	6.5±0.6	5.7±0.5	5.4±0.6	5.9±0.8	**NS**	**NS**	F = 4.67; p = 0.038	p = 0.001
Rectum	8.3 ±0.3	7.1±1.2	7.6±1.3	8.3±0.4	**NS**	**NS**	F = 6.39; p = 0.017	
**Submucosa thickness (μm)**								
Posterior gut	34.8±0.8	34.4±3.0	36.1±2.0	34.1±0.4	**NS**	**NS**	**NS**	p = 0.001
Rectum	25.3±5.0a	19.2±0.9c	24.0±4.9a	21.4±3.3b	F = 51.31; p = 0.001	**NS**	F = 6.09; p = 0.014	

Diets: C (control diet), GMOS (5000 ppm galactomannan oligosaccharides), PHYTO (200ppm phytogenic), GOSPHYTO (5000 ppm galactomannan oligosaccharides+200ppm phytogenic). Posterior gut (preileorectal valve segment) and rectum (postileorectal valve segment) as detailed in Fig 1. Data presented as mean ± SD. N = 4x3 (fish x tank). Different letters within a row denote significant differences among dietary treatments (p≤0.05; one-way ANOVA; Tukey). Two-way ANOVA analyses (p<0.05). NS = No significant. Quantitative differences between posterior and rectum segments, were calculated using T student or U Mann-Whitney tests (p<0.05).

**Fig 4:**
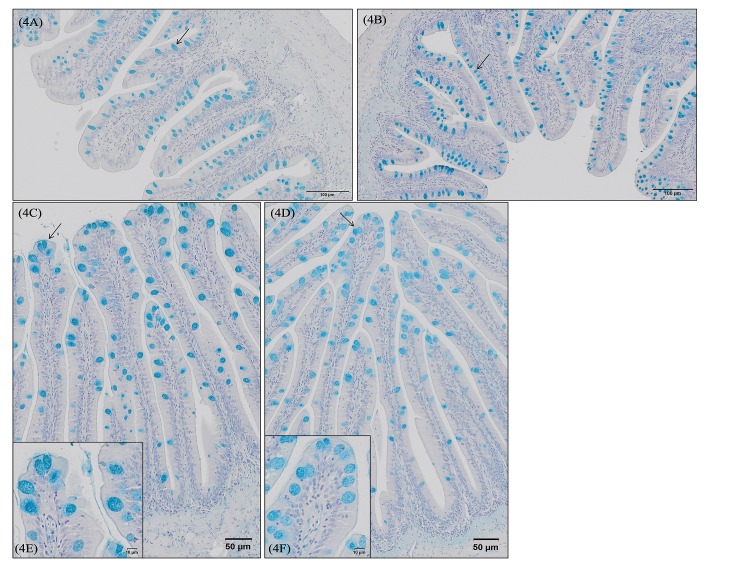
European sea bass (*Dicentrarchus labrax*) posterior gut and rectum goblet cell size and density after 63 days of dietary supplementation. For posterior gut (preileorectal valve), the larger goblet cells and lower density pattern is represented in Fig 4A and corresponds to fish fed control diet. Bar 100 μm. The smaller goblet cells and higher density pattern is represented in Fig 4B and corresponds to fish fed GMOS and PHYTO diets. Bar 100 μm. For rectum (postileorectal valve), the larger goblet cells and lower density pattern is represented in Fig 4C and 4E and corresponds to fish fed control diet. Bar 50 μm and 10 μm. The smaller goblet cells and higher density pattern are represented in Fig 4D and 4F and correspond to fish fed GMOS diets. Bar 50 μm and 10 μm. No variations were found in the % of mucosal area covered by mucus among the dietary treatments for both intestinal segments. C (control diet), GMOS (5000 ppm galactomannan oligosaccharides), PHYTO (200ppm phytogenic), GMOSPHYTO (5000 ppm galactomannan oligosaccharides+200ppm phytogenic).

**Fig 5:**
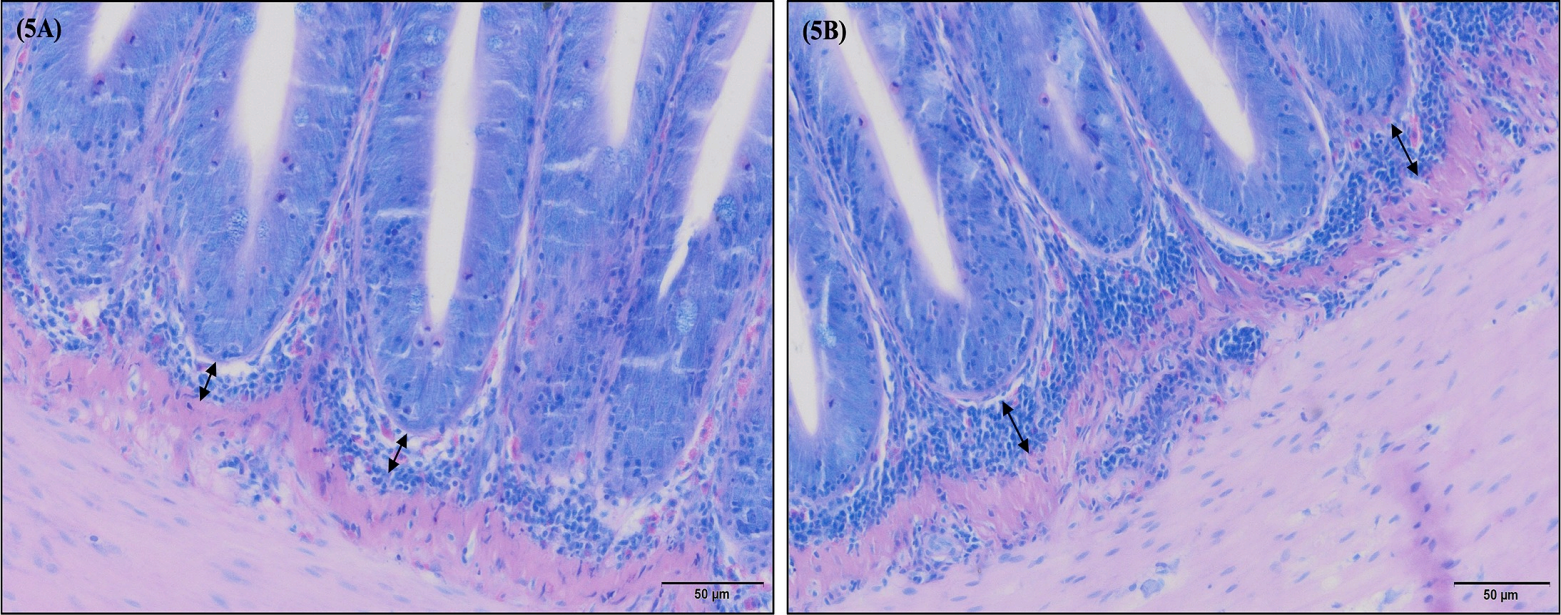
European sea bass (*Dicentrarchus labrax*) rectum submucosa thickness after 63 days of dietary supplementation (May-Grünwald Giemsa). The thinner rectum submucosa pattern observed is represented in Fig 5A and corresponds to fish fed GMOS and GMOSPHYTO diets. Bar 50 μm. The engrossed rectum submucosa pattern observed is represented in Fig 5B and corresponds to fish fed control and PHYTO diets. C (control diet), GMOS (5000 ppm galactomannan oligosaccharides), PHYTO (200ppm phytogenic), GMOSPHYTO (5000 ppm galactomannan oligosaccharides+200ppm phytogenic).

Evaluation of morphometrical characteristics in relation to the dietary treatment revealed no differences (p>0.05) in mucosal fold length. However, fish fed the control diet showed the highest percentage of fold area covered by mucus compared to fish fed the other diets (Table 6). Additionally, functional additives affected the morphological characteristics of goblet cells. For posterior gut, fish fed GMOS and PHYTO diets presented smaller (p<0.05) goblet cells than fish fed the control diet (Fig 4A *vs* 4B), whereas for the rectum segment, only fish fed GMOS diet presented reduced (p<0.05) goblet cell area in comparison to fish fed the control diet (Table 6, Fig 4C *vs* 4D). Despite these differences, dietary treatments did not affect (p>0.05) the pattern of goblet cell distribution along the folds in posterior gut or rectum segments, where the highest goblet cells density was observed in apical and mid regions rather than in the basal zone, as described in Fig 3B. No differences (p>0.05) were found in minimum cell diameter in the two intestinal segments studied (Table 6). Fish fed GMOS and GMOSPHYTO diets presented a thinner (p<0.05) rectum submucosa than fish fed the control and PHYTO diets (Table 6, Fig 5A *vs* 5B); however, no differences were observed in posterior gut segment for this parameter. Accordingly, two-way ANOVA analyses revealed a reducing effect of GMOS (F = 14.53; p = 0.001) and PHYTO (F = 5.52; p = 0.019) in terms of percentage of fold coverage, as well as a reducing effect of PHYTO (F = 3.95; p = 0.049) diet on the size of the posterior gut goblet cells. Dietary GMOS supplementation resulted in a reduction in rectum submucosa thickness (F = 51.31; p = 0.001). A significant interaction between GMOS and PHYTO diet factors was detected for posterior gut and rectum goblet cell area (F_posterior_ = 8.73; p = 0.006; F_rectum_ = 10.05; p = 0.003), diameter (F_posterior_ = 4.67; p = 0.038; F_rectum_ = 6.39; p = 0.017), and rectum submucosa thickness (F_rectum_ = 6.09; p = 0.014) (Table 6).

**Fig 6:**
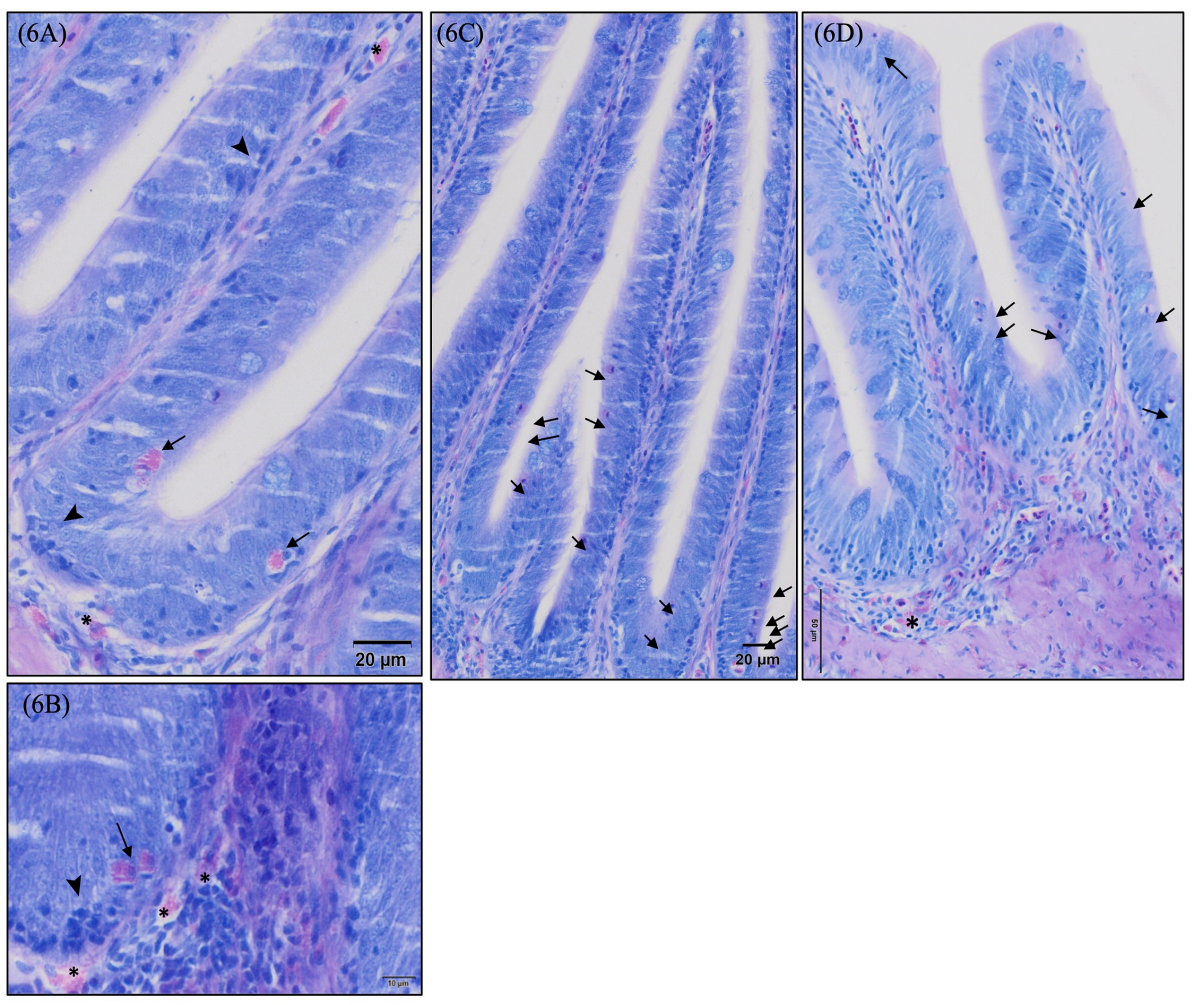
Detailed micrographs of infiltrated granulocytes and lymphocytes and rodlet cells present in European sea bass (*Dicentrarchus labrax*) intestinal mucosa stained with May-Grünwald Giemsa. (6A) Detail of infiltrated granulocytes in the rectum mucosa (→), submucosa and lamina propia (*). Infiltrated lymphocytes are indicated by ➤. Scale bar 20 μm. (6B) Detail of infiltrated granulocytes (→) and lymphocytes (➤) in posterior gut mucosa. Observe the mixed population of leukocytes in the submucosa. Granulocytes are indicated by (*). Scale bar 10 μm. Observe the higher density of rodlet cells present in the basal region of the fold compared to the apical region in rectum (6C) and posterior gut (6D). Scale bar 50μm- 20 μm.

The degree of leukocytes (granulocytes and lymphocytes) present in *lamina propria* and infiltrated in the intestinal mucosa was similar in both intestinal segments, except for the number of granulocytes at submucosa, which was greater in posterior gut than in the rectum. Fig 6A and 6B show details of infiltrated leukocytes. The degree of granulocyte and lymphocyte infiltration for both intestinal segments was not affected (p>0.05) by dietary treatments (Table 7).

**Table 7:** Scoring of submucosa-and *lamina propria-*infiltrated leukocytes subpopulations in posterior gut (preileorectal valve) and rectum (postileorectal valve) for European sea bass (*Dicentrarchus labrax*) at the end of the feeding trial (t = 63 days).

			Dietary Treatments				Two-way ANOVA		
			Control	GMOS	PHYTO	GMOSPHYTO	GMOS	PHYTO	GMOS*PHYTO
Posterior gut	Granulocytes	Submucosa	2–3	2–3	2	2–3	NS	NS	NS
		Lamina propia	1–2	1–2	1–2	1–2	NS	NS	NS
	Lymphocytes	Submucosa	2–3	2–3	2–3	2–3	NS	NS	NS
		Lamina propia	2–3	2–3	2–3	2–3	NS	NS	NS
	Rodlet cells		1–2	1–2	2	1–2	NS	F = 3.604; P = 0.068	F = 3.398; P = 0.076
Rectum	Granulocytes	Submucosa	2	2	2	1–2	NS	NS	F = 4.641; P = 0.040
		Lamina propia	1–2	1–2	2	1–2	NS	NS	NS
	Lymphocytes	Submucosa	2–3	2	2	2	NS	NS	NS
		Lamina propia	2	2	2	2	NS	NS	NS
	Rodlet cells		2	3	2–3	2–3	F = 5.946; P = 0.020	NS	NS

ND (not detected), 1 (low), 2 (moderate) and 3 (high) for each area/cell evaluated.

Posterior gut presented a lower density of rodlet cells as compared to rectum; however, distribution in the two segments was similar, being more concentrated in the basal zone of the folds than in the apical region (Fig 6C and 6D). Two-way ANOVA detected a trend for an increased number of rodlet cells in fish posterior gut after dietary PHYTO supplementation (F = 3.604; p = 0.068), whereas in rectum, this effect was attributed to dietary GMOS (F = 5.946; p = 0.022). A significant interaction between GMOS and PHYTO factors was detected for the presence of posterior gut rodlet cells (F = 3.398; p = 0.076) (Table 7).

#### Posterior gut and rectum structure study

**Fig 7:**
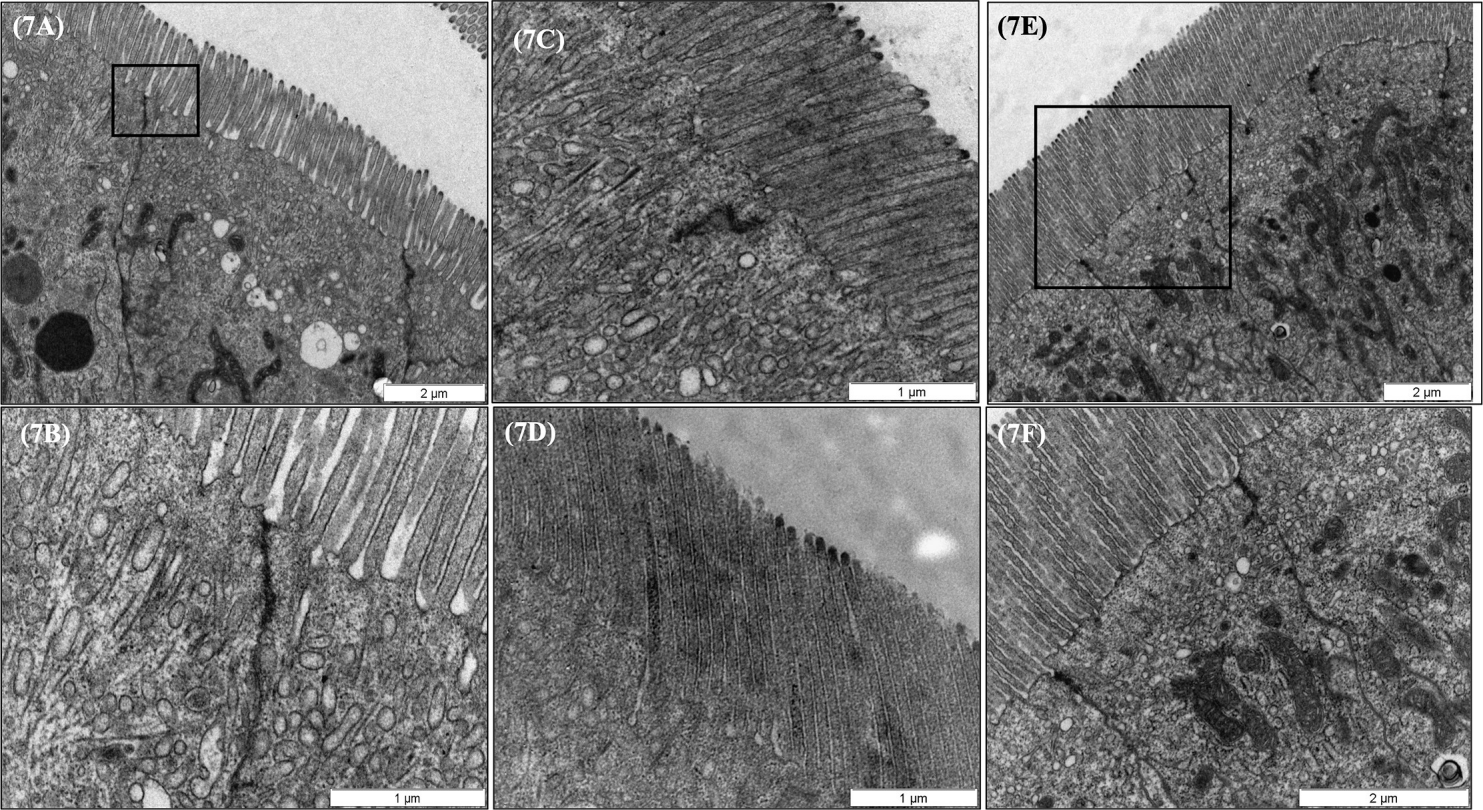
Representative TEM micrographs of the posterior and rectum intestinal regions of European sea bass (*Dicentrarchus labrax*) fed the different dietary treatments. (7A) Posterior gut structural pattern observed for all fish studied. Scale bar 2 μm. (7B) Detailed micrograph of posterior gut tight junction (TJ) appearance. Observe the conserved structure. Scale bar 1μm. (7C) Pattern of rectum microvilli length for fish fed control and PHYTO diet and (7D) representative micrograph of rectum microvilli length for fish fed GMOS based diets which were significantly (P<0.05) longer than fish fed the rest of the dietary treatments. Scale bars 1μm. No evident differences were observed in enterocyte packaging, membrane lining appearance (7E), or TJs structure (7F) among dietary treatments for rectum region. Scale bars 2 μm. C (control diet), GMOS (5000 ppm galactomannan oliGMOSaccharides), PHYTO (200ppm phytogenic), GMOSPHYTO (5000 ppm galactomannan oligosaccharides+200ppm phytogenic).

Observations in the preileorectal (posterior gut) and postileorectal (rectum) regions refer to European sea bass fed the experimental diets for 63 days. For posterior gut, the qualitative TEM revealed a similar morphological pattern for all fish, regardless of the dietary treatment, in terms of membrane lining appearance, cytoplasmic electron density, enterocyte packaging, TJ structure, and level of infiltrated leukocytes (Fig 7A and 7B). For the rectum region, the structural study revealed increased microvilli length (p<0.05) for fish fed GMOS diets (mean length (μm) = 2280.95 ± 156.86; max length = 2584.91 μm; min length = 1997.90 μm) in comparison to fish fed control and PHYTO diets (mean length (μm) = 1541.44 ± 150.42; max length = 1783.67 μm; min length = 1380.83 μm) (Fig 7C and 7D). However, despite the differences found in microvilli length, all analyzed fish presented a normal microvillar morphology of nondamaged and well- packaged enterocytes and conserved TJ structure and membrane lining appearance (Fig 7E and 7F).

**Fig 8:**
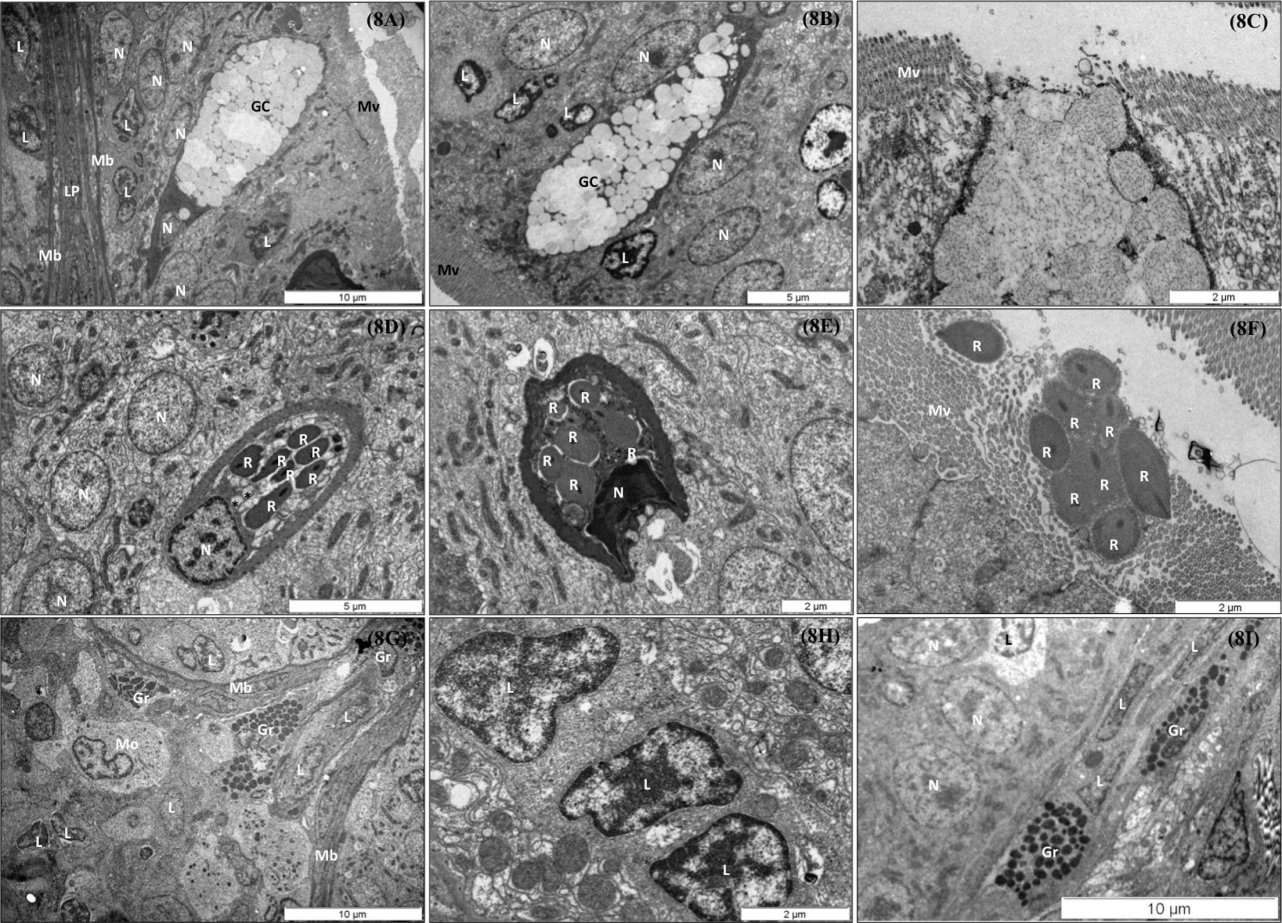
Detailed TEM micrographs of goblet cells, rodlet cells, lymphocytes, and eosinophilic granule cells (granulocytes) housed in the gut of European sea bass (*Dicentrarchus labrax*) juveniles. (A) Goblet cells scattered among enterocytes and characterized by a clearly polarized shape with narrow base containing the nucleus and organelles and an expanded apical region containing the secretory granules; observe granules with different electron density. Scale bar 10 μm. (B) Goblet cells surrounded by lymphocytes. Scale bar 5 μm. (C) Detail of goblet cell in process of mucus exocytosis inside the lumen. Scale bar 2 μm. (D) Intraepithelial immature rodlet cell characterized by a single nucleus with condensed heterochromatin at nuclear periphery and a cytoplasm containing translucent vesicles (*) and rodlets (R) with electron dense cores. Orientation in parallel to the surface layer of the epithelium. Scale bar 5 μm. (E) Intraepithelial mature rodlet cell oriented perpendicular to the surface layer of the epithelium. Scale bar 5 μm. (F) Free rodlet bundles in the microvilli. Scale bar 2 μm. (G) Representative micrograph of leukocytes housed in the intestinal submucosa. Scale bar 10 μm. (H) Detail of lymphocytes characterized by a single nucleus with areas of condensed heterochromatin. Scale bar 2 μm. (I) Representative micrograph of granulocytes housed in the lamina propia. Scale bar 10 μm. Abbreviations used are: GC: Goblet cell; Gr: granulocyte; L: Lymphocyte; LP: Lamina propria; Mb: Basal membrane; Mo: Macrophage; Mv: Microvilli; N: Nucleus; R: Rodlet.

By using TEM analyses, we could to clearly observe the morphology of goblet cells scattered among enterocytes: a clearly polarized shape characterized with a narrow basal region containing the nucleus and organelles and an expanded apical region containing the secretory granules, which had a different electron density (Fig 8A). Goblet cells were normally in contact with lymphocytes, not only in the basal area but also in the apical region (Fig 8A and 8B). Fig 8C shows a goblet cell in the exocytosis process, secreting mucus to the lumen. Furthermore, we could identify immature (Fig 8D) and mature rodlet cells (Fig 8E) scattered among enterocytes in the basal fold region by using TEM. Intraepithelial immature rodlet cells were characterized by a single nucleus with condensed heterochromatin at nuclear periphery and a cytoplasm containing translucent vesicles. Rodlet cells with electron- dense cores were oriented in parallel to the surface layer of the epithelium (Fig 8D), whereas mature rodlet cells were oriented perpendicular to the surface layer of the epithelium (Fig 8E) and the vesicles had a greater electron density. Fig 7F shows discharged rodlets within the microvilli.

TEM structure analysis showed the presence of infiltrated leukocytes in the intestinal mucosa, *lamina propria*, and submucosa (Table 7; Fig 8G). As reported in Table 7, the main cell type of infiltrated leukocytes in European sea bass mucosa corresponded to the structural characteristics of lymphocytes (Fig 8H), which were frequently in contact with granulocytes (Figs 8G and 7I) and macrophages (Fig 8G).

#### Gene expression analyses

**Fig 9:**
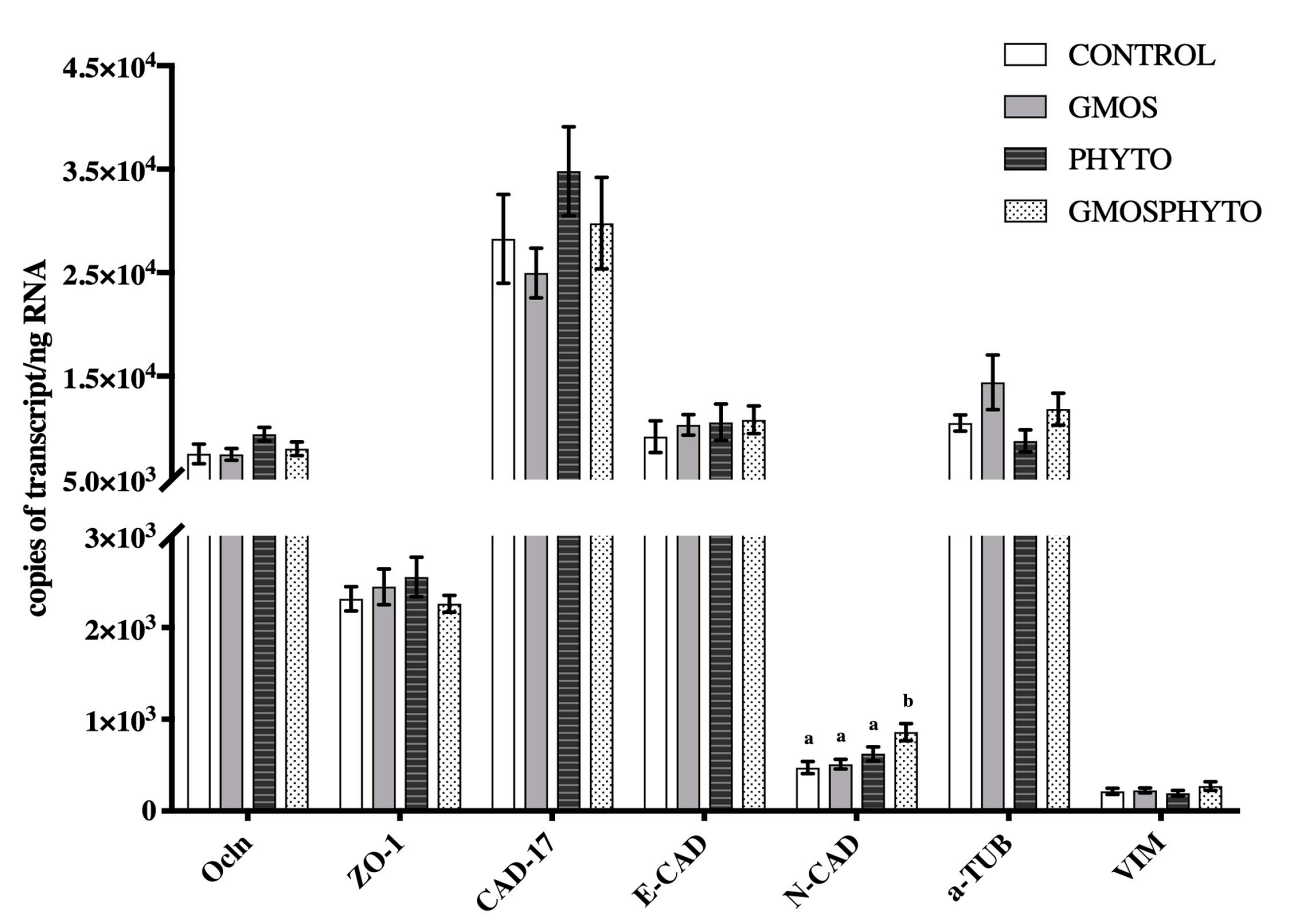
Absolute quantification of transcript copies of *Ocln*, *ZO-1*, *Cad-17*, *E-Cad*, *N-Cad*, *a-Tub*, *and vim* genes in European sea bass (*Dicentrarchus labrax*) posterior gut (mean ± SEM, n = 6 fish/diet). Functional low FM and FO diets were fed for 63 days. C (control diet), GMOS (5000 ppm galactomannan oligosaccharides), PHYTO (200ppm phytogenic), and GMOSPHYTO (5000 ppm galactomannan oligosaccharides+200ppm phytogenic). Different letters denote significant differences among dietary treatments (p≤0.05; one-way ANOVA; Tukey). Two-way ANOVA analyses found an increased (p≤0.10) absolute quantification of transcript copies for *Ocln*, *N-Cad*, and *Cad-17* to PHYTO dietary supplementation in low FM and FO diets at the end of the feeding trial (F_ocln_ = 3.299; p = 0.87; F_N-Cad_ = 15.861; p = 0.001; F_Cad-17_ = 2.415; p = 0.130).). For detailed information see supporting information S1 Fig.

Fig 9 shows the transcript levels of tight junction (*Ocln*, *ZO-1*), adherens (*Cad-17*, *E-Cad*), desmosomes (*N-Cad*), and cytoskeletal (*α-Tub* and *Vim*) genes in the posterior gut of European sea bass at the end of the feeding trial (63 days). One-way ANOVA analyses showed no influence of dietary treatment on the mRNA copies of *Ocln*, *ZO-1*, *Cad-17*, *E-cad*, *α-Tub*, and *Vim* genes. In contrast, the expression level of *N-Cad* was increased (p<0.05) by GMOSPHYTO diet supplementation (Figs 9 and S1 Fig).

Two-way ANOVA analyses found a statistically significant increase (p≤0.10) in transcript copies for *Ocln*, *N-Cad* and *Cad-17* to PHYTO diet at the end of the feeding trial (F_ocln_ = 3.299; p = 0.87; F_N-Cad_ = 15.861; p = 0.001; F_Cad-17_ = 2.415; p = 0.130). No significant effect due to GMOS supplementation or to the interaction between GMOS and PHYTO factors was detected.

### Experiment II: Intestinal infection and stress challenge

#### Relative percentage of survival RPS

PHYTO and GMOS dietary supplementation for 63 days reduced European sea bass susceptibility to *V*. *anguillarum* after intestinal infection combined with stress by confinement for 7 days. The RPS values for the dose inoculated was 33%, 47% and 20% for fish fed GMOS, PHYTO, and GMOSPHYTO, respectively. Fish fed control died achieved a final mortality of a 40%.

#### Goblet cell morphology and mucus production patterns during the challenge test. A time course study

**Fig 10:**
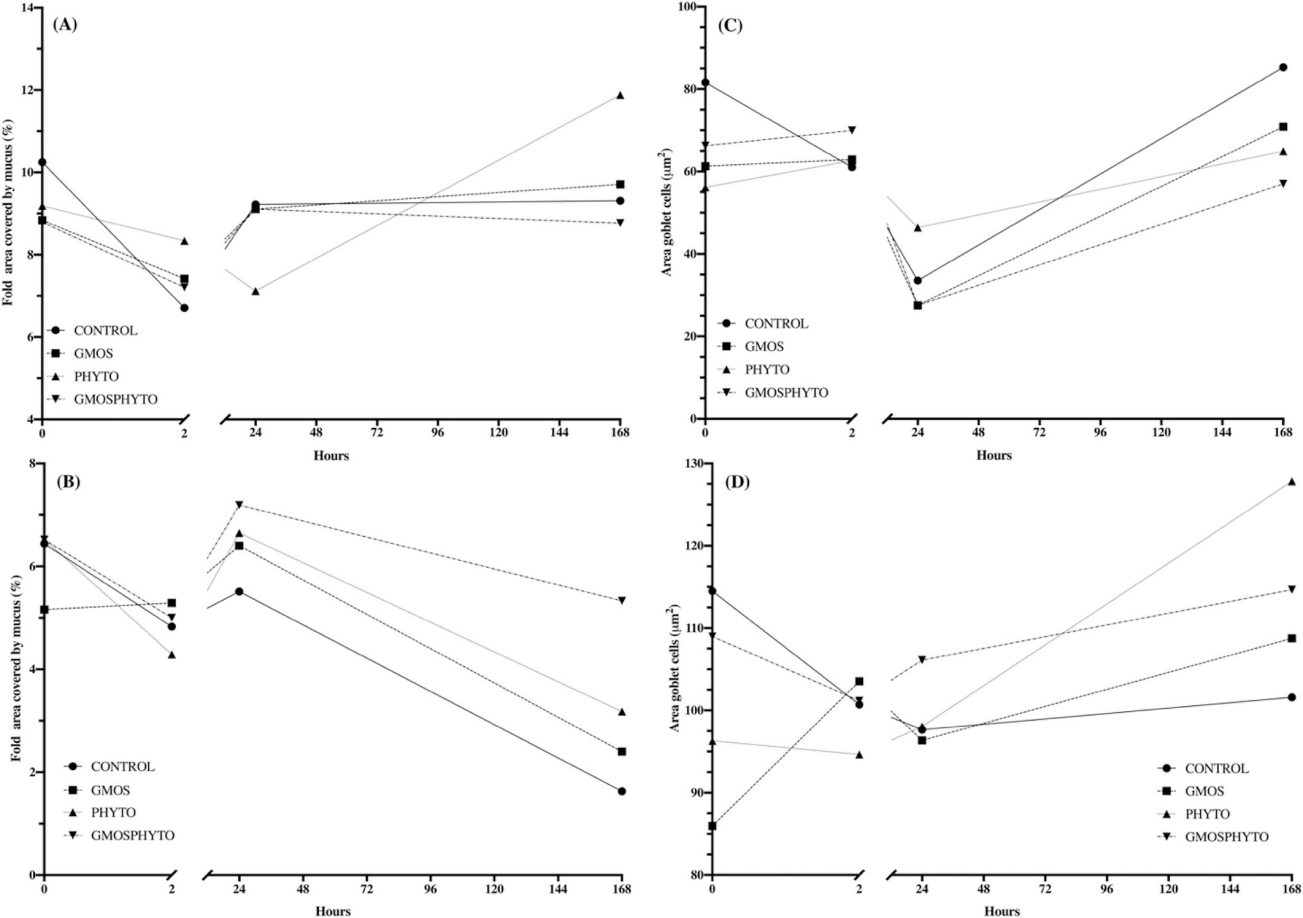
Time course study of European sea bass (*Dicentrarchus labrax*) posterior and rectum gut fold area percentage covered by goblet cells and goblet cell area (μm^2^) during the 7 days of the challenge test (*V*. *anguillarum* intestinal infection + stress by confinement). Functional low FM and FO diets were fed for 70 days (63 feeding+7days of challenge test). (A) Posterior gut fold area percentage covered by goblet cells during the challenge test. Observe the different pattern of response presented by fish fed PHYTO diet, which shows a trend for a higher percentage of mucus coverage after 2h of challenge, which is reduced after 24h, and clearly recovered after 7 days of challenge test compared to fish fed other dietary treatments. (B) Rectum fold area percentage covered by goblet cells during the challenge test. Observe the different pattern of response presented by fish fed GMOSPHYTO diet, which show a higher percentage of mucus coverage 24h and 7 days (p<0.05) after challenged compared to fish fed control diet. Two-way ANOVA analyses showed an increased percentage of fold covered by mucus for PHYTO dietary supplementation in low FM and FO diets at the end of the challenge test (F = 12.56; p = 0.012). (C)Posterior gut goblet cells area (μm^2^) during the challenge test. Observe how initial patterns are recovered after 7 days of challenge test for all dietary treatments, fish fed control diet having larger goblet cells (p<0.05) than fish fed PHYTO diet. However, 24h post challenge fish fed PHYTO diet presented larger (p<0.05) goblet cells than fish fed GMOS and GMOSPHYTO diets. Two-way ANOVA analyses associated a significant reduction in goblet cell size to dietary PHYTO supplementation in low FM and FO diets for 63 days (t = 0 hours; F = 3.95; p = 0.05) as well as at the end of the challenge test (t = 168 hours; F = 6.94; p = 0.030). On the other hand, PHYTO dietary supplementation in low FM and FO diets was associated with an increase in goblet cell size (F = 7.67; p = 0.013) 24h after the challenge. GMOS supplementation was associated with a reduction in goblet cell size 24h (t = 24 hours; F = 7.676; p = 0.013) and 7 days after the challenge (t = 168 hours; F = 16.26; p = 0.004). (D) Rectum goblet cell area (μm^2^) during the challenge test. Observe how fish fed PHYTO diet presented a larger (p<0.05) goblet cell area than fish fed control diet at the end of the challenge test (t = 168h). C (control diet), GMOS (5000 ppm galactomannan oligosaccharides), PHYTO (200ppm phytogenic), GMOSPHYTO (5000 ppm galactomannan oligosaccharides+200ppm phytogenic). For detailed information see supporting information S2 Fig.

No significant differences (p>0.05, N = 9 fish diet) were found for the percentage of posterior gut fold area covered by goblet cells during the challenge test. However, fish fed PHYTO diet presented a clearly different pattern of response to the experimental intestinal inoculation of bacteria in comparison to fish fed the other two diets (Fig 10A).

As can be seen in Fig 10A (for detailed information see S2 Fig), fish fed PHYTO diet presented a trend for having the highest percentage of mucus coverage after 2h of challenge, which was reduced after 24h. Then, after 7 days of challenge test, mucus production had clearly recovered in comparison to fish fed the other diets, thus responding in a more controlled and delayed manner way to the external stressor. Indeed, at 24h after the stress, in fish fed PHYTO diet, despite showing a trend for having the lowest percentage of area covered by mucus, goblet cells were larger than in fish fed the other diets, confirming this tendency (Fig 10C and S2 Fig). In the case of rectum section, fish fed GMOSPHYTO diet presented higher (p<0.05) fold mucus coverage 7 days after being challenged in comparison to fish fed the control diet (Fig 10B and S2 Fig). Two-way ANOVA analyses associated this effect with PHYTO dietary supplementation (F = 12.56; p = 0.012).

With regard to the posterior gut’s goblet cell area (μm^2^), fish fed PHYTO diet had larger (p<0.05) goblet cells than fish fed GMOS and GMOSPHYTO diets at 24h after the challenge. Initial goblet size patterns returned then to normal size after 7 days of challenge test in fish from all dietary treatments, with fish fed control diet having larger goblet cells (p<0.05) than fish fed PHYTO diet (Fig 10C and S2 Fig). Two-way ANOVA analyses associated a significant reduction in goblet cell size with dietary PHYTO supplementation both before the challenge period (t = 0 hours; F = 3.95; p = 0.05) and at the end of the challenge test (t = 168 hours; F = 6.94; p = 0.030). Similarly, GMOS supplementation was associated with a reduction in goblet cell size at 24h (t = 24 hours; F = 7.676; p = 0.013) and at 7 days after the challenge (t = 168 hours; F = 16.26; p = 0.004). As for rectum, at the end of the challenge test (t = 168h), goblet cell areas were larger (p<0.05) in fish fed PHYTO diet than fish fed control diet (Fig 10D and S2 Fig).

## Discussion

Fish feeding behavior was monitored during the feeding trial. In this time, neither was a perceptible behavioral change nor were any appetite alterations observed. Dietary GMOS (Galactomannan oligosaccharides from mucilage) or/and PHYTO (mixture of garlic oil and labiatae plants oils) did not affect European sea bass growth performance or feed utilization after 63 days of feeding, being adequate and similar to that reported previously for the same fish species and diet composition (10%FM/6%FO) [42]. Fish studies with prebiotics and phytogenics, in general have been addressed to promote fish health and disease resistance [for review see 1, 32, 43–44]. Indeed, both functional additives used in the present study significantly improved European sea bass disease resistance against *V*. *anguillarum* after experimental intestinal infection.

GMOS functions as a dietary fiber due to its indigestibility [45]; its protective effect in the present experiment is probably related to a direct prevention of bacterial attachment to the IECs through its mannose units, via adhering to bacterial pathogen receptors [46] and avoiding the first step of intestinal *V*. *anguillarum* colonization after inoculation. However, due to its composition rich in mannose and galactose, a general stimulation of the systemic and gut associated lymphoid tissue (GALT) may also help reduce disease incidence. Indeed, there is evidence of enhanced immune response in other fish mucosal tissues, such as skin, in sea bass and other Mediterranean fish species after feeding with a rich GMOS product from several sources [47–49].

In the case of PHYTO diet, there are several previous studies that relate some of its bioactive compounds to fish enhanced disease resistance, too. However, most of these studies have been addressed to fresh water species fed with standard diets formualtions. For example, both fresh oil and powdered forms of garlic (*Allium sativum*) have been proven to be an effective prophylactic and therapeutic agent in fresh water species [49–58], which effects on fish survival are dependent on the percentage or dosage supplemented, as demostrated also in marine species such as European sea bass [55]. Its protective effects have been attributed to a general enhancement of the immune system [53–54, 58–59] and antioxidant responses [60–63]. Likewise, garlic dietary administration has been associated with changes in the intestinal microbiota, conferring beneficial effects to the host [64]. Oil and powdered forms of Labiatae plants species such as rosemary (*Rosmarinus officinalis*) or thyme (*Thymus vulgaris*) have been also related with enhanced fish immune response [65–66] and disease resistance [29, 67–69]. Similarly, a dietary combination of oregano (*Origanum heracleoticum*), carvacrol, and thymol increased resistance to *A*. *hydrophila* in channel catfish (*Ictalarus punctatus*) [70].

The mechanisms of action of phytogenics in fish disease resistance will depend on their chemical composition [71, 32] and several reviews focused mainly in continental aquaculture are currently available [32, 50, 72]. In general terms, however, the antimicrobial mode of action relies on altering bacterial cell permeability/fluidity and quorum sensing systems [32,71,73]. In addition, other recognized effects of phytogenics in animal production are the reduction in gut oxidative stress, stabilization of intestinal microbiota, and modulation of the immune system via scavenging of free radicals [32,74].

In the present study, the pathological appearance and subsequent fish death were conditioned by a successful gut translocation of intestinally inoculated *V*. *anguillarum*, which had to cross the gut extrinsic and physical barriers to first get in touch with the GALT and, then, to arrive in internal organs. Gut bacterial translocation rates were clearly reduced in fish fed functional diets, but the morphological and morphometrical studies carried out indicated that the effects observed are region and additive specific.

Two regions of the European sea bass gut were studied, the preileorectal (posterior gut) and postileorectal valve (rectum) regions, which present a clearly different morphology, the latter with a wider *muscularis* layer, longer diameter and folds, and thinner submucosa than posterior gut [75–76]. Additionally, posterior gut showed greater mucus coverage because of an increased cell density of smaller goblet cells located on lower fold regions (mid and basal), whereas goblet cells in the rectum are larger and mainly located on upper fold regions (apical).

No effect of diet was observed in fold morphometric characteristics or in goblet cell distribution patterns; however, fish fed the non-supplemented diet showed larger goblet cells than fish fed functional diets. Indeed, PHYTO supplementation reduced posterior gut goblet cell size, whereas GMOS reduced the goblet cell area in posterior gut and rectum and the submucosal width in the rectum region, indicating overall to a site- and product-specific anti-inflammatory role of the two products. However, a combination of the two was not that effective, pointing to an antagonistic effect of these products.

Previous studies in European sea bass fed similar levels of VM/VO, reported for the posterior gut increased density of goblet cells and up-regulated IL-1ß, tumor necrosis factor α (TNFα), and cyclooxygenase-2 (COX2) gene expression in relation to a general gut inflammation-like status caused by alterations in the composition of autochthonous microbiota [23–24]. Indeed, in higher vertebrates, increased levels of IL-1ß and TNFα have been associated with augmented mucosal secretions [77–80] and changes in mucin composition [80]. In Atlantic salmon, intestinal VM-induced enteritis is mediated by T cells [81–83], as occurs with intestinal goblet cell hyperplasia and alterations in mucus production/composition rates in mammals after exposure to external insults [80, 84–86]. Despite the lack of any evident effect in the present study of PHYTO or GMOS supplementation on the incidence of submucosa and *lamina propia* infiltrated leucocytes (lymphocytes and granulocytes) on both intestinal regions studied, a possible change on the T cells populations ratio derived from their supplementation should be considered. In particular, those populations involving T-cell activation via specialized antigen-presenting cells (APC) should be considered since feeding low FM levels in European sea bass juveniles has been related to an upregulation of MHCII gene expression [23]. Interestingly, the structural studies carried out in the present investigation revealed that goblet cells of both intestinal regions are regularly in contact with lymphocytes. In higher vertebrates, goblet cells deliver luminal antigens to APC (CD103+ dendritic cells) of the *lamina propria* [87], which are involved in a tolerance mechanism mediated by T cells [88–89], although in fish this pathway has not been described in fish.

In this sense, GMOS, despite its function to prevent bacterial attachment to the IECs, may affect positively European sea bass intestinal mucosal health- through its role as dietary prebiotic- by balancing the low dietary FM/FO-associated dysbiosis, particularly in rectum, as denoted by its effect on the submucosal inflammatory status, the reduced goblet cell hyperplasia, and the increased microvilli height. A similar beneficial, cytoprotecting effect on gut epithelium has been described in sea bass and other fish species fed similar prebiotics [for review see 43, 90–91]. Particularly, GMOS as an indigestible fiber may serve as a substrate to the rectum microbiota as it does for the mucus layer [92], promoting beneficial bacterial growth and short-chain fatty acids production [93–94], as opposed to favoring bacteria capable of degrade mucins and disrupt the epithelial layer [95]. Furthermore, there is evidence of a systemic and local immune system stimulation after feeding GMOS-based products to Mediterranean fish species [47–49], which may contribute to the reduced disease incidence found.

Actually, after *V*. *anguillarum* gut inoculation, fish fed GMOS presented a similar pattern of mucus release/production response to fish fed control diet for posterior gut but differed for rectum from other treatments. The posterior gut mucus response to *V*. *anguillarum* and confinement was characterized by: (a) an immediate (2h) reduced mucus coverage as a direct consequence of mucus discharge after bacterial inoculation in order to rapidly wash away inoculated *V*. *anguillarum*; (b) an increase in mucus coverage after 24h as a result of higher density of smaller goblet cells, indicating goblet cells proliferation as a mid-term protection mechanism and (c) recovery to initial values after 7 days of challenge. Instead, for the rectum, fish fed GMOS presented increased goblet cell size 2h after inoculation compared to basal size, differing from the rest of the treatments and probably indicating variations in mucus composition via new mucin synthesis and granule storage, before being released in a more controlled way in response to the pathogen presence. Particularly in fish, the timing and precise mechanisms regulating mucin production and goblet cell secretion after infection remain unknown [80]. However, in the outer mucus layer of human colon, it is known that MUC2 mucin assembly and granular accumulation to replenish new goblet cells takes longer than 4-5h [89], which, if similar in fish, could support the hypothesis of new mucin synthesis and storage before release in fish fed GMOS diet.

In the case of PHYTO, its protective role seems to be limited to the pre-ileorectal valve segment, where clearly there was a reduced goblet cell size as compared to fish fed control diet, and it is also likely involved in buffering the effects of low FM/FO diets on the posterior gut microbiota populations. Phytogenics are recognized reducing agents for gut oxidative stress, stabilizing microbiota via altering quorum sensing of pathogenic bacteria and modulating the local immune system via scavenging of free radicals [32,74]. Indeed, a parallel study using the same dietary phytogenic mixture supplementation (PHYTO) mitigated European sea bass stress response and reinforced the systemic immune system response in stressed and infected fish by protecting leukocytes of apoptotic processes associated to stress [49]. This process may be helping fish fed PHYTO to: (a) reduce gut bacterial translocation rates after inoculation as cortisol increases have been associated with increased paracellular permeability in several species [96–98]; and (b) reduce reactive oxygen species (ROS) causing IEC damage via enzymatic control of ROS generation in cellular responses to cytokines and bacterial invasion [99]. Indeed, feeding high levels of terrestrial ingredients has been related to a damaged intestinal barrier in several fish species [24,100–101]. Damage-associated molecular patterns released from necrotic, apoptotic, or damaged IECs trigger the release of several proinflammatory cytokines that, in turn, may alter mucus composition and the abundance of goblet cells [24,102–103] or affect epithelial barrier maintenance. Actually, posterior gut of fish fed PHYTO presented a general trend, although not statistically significant, for an increased number of *Ocln*, *zo-*1, *cad-17*, and *N-cad* transcript copies, which may be involved in the lower paracellular *V*. *anguillarum* translocation rate found after the inoculation + confinement challenge. However, this trend was not clearly noticeable at the electron microscopy level. A similar trend has been observed in broiler chickens fed an *Allium hookeri-* based phytogenic, which showed improved gut barrier function in relation to upregulation of gut TJ related genes [74]. In addition to any possibly protective epithelial barrier effect, garlic and labiatae plant extracts have been demonstrated to be effective as dietary immunostimulants in European sea bass and other fish species [49–51, 55, 69, 104–106], which may help to fight against translocated *V*. *anguillarum* as compared to non-supplemented fish.

Interestingly, the same intestinal regions especially affected by GMOS and PHYTO in terms of goblet cell size reduction or reduced inflammation presented a higher density of rodlet cells scattered in the basal fold region. Very little is known about their role in fish mucosal immunity and traditionally their presence has been related to exposure to infectious processes/noxious agents/stressors [107–111] and the associated tissue injuries [112]. An antibiotic nature of their secretory product has also been proposed, supported by their positivity for alkaline phosphatase, peroxidase, melatonin stimulation hormone, and the antimicrobial peptide piscidin [113], which in the present study may have been stimulated via a microbiota composition modulation after supplementation with functional additives. Further studies are being conducted along this line. Indeed, these studies will help to clarify the results obtained in the case of fish fed the GMOSPHYTO diet, pointing to an antagonistic effect of the simultaneous supplementation of the two products on the European sea bass gut mucosal defense system.

Our results indicated that the supplementation of GMOS (5000 ppm) and PHYTO (200ppm) in 10%FM/6%FO based diets affects European sea bass gut health maintenance in a site-specific way, GMOS having a greater effect along the second segment of the gut and PHYTO effect being focused on the preileorectal valve region. These protective effects, among others, may contribute to the reduced “*in vivo*” *V*. *anguillarum* bacterial translocation rates found in terms of higher survival after being intestinally inoculated and combined with a panel of stress by confinement. Both supplements are emerging as potential functional products for gut health maintenance when diets with low levels of ingredients of marine origin are fed and in the dosage supplemented, and further studies are being conducted in order to establish which modes of action are being affected by each product on each specific intestinal region in terms of GALT and stress response, as well as their effects on the European sea bass microbiome, this being the first of a series of publications on this topic.

## Supporting information

S1 Fig Gene expression data for posterior gut genes (*ocln*, *zo-1*, *cad-17*, *e-cad*. *N-cad*, *a-Tub*, *vim*) analysed by One-step TaqMan® real-time RT-PCR. (XLSX)

S2 Fig Time course study data of European sea bass (*Dicentrarchus labrax*) posterior and rectum gut fold area percentage covered by goblet cells and goblet cell area (μm2) during the 7 days of the challenge test (*V*. *anguillarum* intestinal infection + stress by confinement). (XLSX)
